# Protective effects of a gastrointestinal agent containing Korean red ginseng on gastric ulcer models in mice

**DOI:** 10.1186/1472-6882-10-45

**Published:** 2010-08-18

**Authors:** Atsushi Oyagi, Kenjirou Ogawa, Mamoru Kakino, Hideaki Hara

**Affiliations:** 1 Molecular Pharmacology, Department of Biofunctional Evaluation, Gifu Pharmaceutical University, 1-25-4 Daigaku-nishi, Gifu 501-1196, Japan

## Abstract

**Background:**

Korean red ginseng (KRG) is a ginseng that has been cultivated and aged for 4-6 years or more, and goes through an extensive cleaning, steaming and drying process. KRG contains more than 30 kinds of saponin components and has been reported as having various biological properties, such as anti-fatigue action, immune restoration, and neurovegetative effect. The purpose of this study was to assess the effects of a KRG-containing drug (KRGCD) on gastric ulcer models in mice.

**Methods:**

Stomach ulcers were induced by oral ingestion of hydrochloride (HCl)/ethanol or indomethacin. Treatment with KRGCD (30, 100, and 300 mg/kg, p.o.) occurred 1 hr before the ulcer induction. Effect of KRGCD on anti-oxidant activity and gastric mucosal blood flow with a laser Doppler flowmeter in mice stomach tissue was evaluated.

**Results:**

KRGCD (100 and 300 mg/kg, p.o.) significantly decreased ethanol- and indomethacin-induced gastric ulcer compared with the vehicle-treated (control) group. KRGCD (100 and 300 mg/kg) also decreased the level of thiobarbituric acid reactive substance (TBARS) and increased gastric mucosal blood flow compared with the control group.

**Conclusions:**

These results suggest that the gastroprotective effects of KRGCD on mice ulcer models can be attributed to its ameliorating effect on oxidative damage and improving effect of gastric mucosal blood flow.

## Background

Korean ginseng (the root of Panax ginseng, C. A. Meyer) has been known to be a valuable and important folk medicine in East Asian countries, including Korea, China, and Japan, for about 2000 years and is now one of the most extensively used botanical products in the world [1,2]. Korean red ginseng (KRG) is a ginseng that has been cultivated and aged for 4-6 years or more, and goes through an extensive cleaning, steaming, and drying process [3]. Among the several kinds of Panax ginseng products, KRG has the most potent multiple pharmacological actions for treating various human diseases, including cardiovascular diseases, rheumatoid arthritis, and diabetes mellitus [4,5].

Gastric ulcer is an illness that affects a considerable number of people worldwide. The etiological factors of this disorder include stress, smoking, alcohol, nutritional deficiencies, infections, and frequent and indiscriminate use of nonsteroidal anti-inflammatory drugs (NSAIDs) [6]. The pathogenesis of gastroduodenal ulcers is influenced by various aggressive and defensive factors, and gastric mucosal blood flow is an important factor regulating the gastric function [7]. A study on the effects of KRG on patients with gastric cancer and post-operative immunity showed that patients treated with KRG had a higher survival rate with complete cure of disease [8]. Previously, the anti-ulcer effects of KRG have been reported in water immersion stress-, serotonin-, and endotoxin-induced gastric ulcer models [9]. On the other hand, the effect of KRG on alcohol- and NSAIDs-induced ulcer models has not been studied.

Ethanol is known to produce erosion, ulcerative lesions, and petechial bleeding in the mucosa of the stomach in humans [10-12]. Ethanol rapidly penetrates the gastric mucosa, and causes membrane damage, exfoliation of cells, erosion, and ulcer formation. Ethanol-induced gastric ulcer models are commonly used to study both the pathogenesis of and therapy for human ulcerative disease [13].

Indomethacin, an NSAID, is widely prescribed in clinical practice because NSAIDs exhibit excellent efficacy in the management of pain, fever, and inflammation through suppression of the synthesis of prostaglandins (PGs) from arachidonic acids resulting from their inhibition of cyclooxygenase (COX) [14]. However, the use of NSAIDs is also associated with significant risks of adverse gastrointestinal events, such as gastric mucosal erosion, ulceration, bleeding, and perforation [15,16].

In this study, we investigated the effects of the KRG-containing drug (KRGCD) on acute gastric injury caused by administration of ethanol or indomethacin in mice.

## Methods

### Animals

Six-week-old male ddY mice weighing 28-30 g were used in this study. The animals were obtained from colonies of specific conventional ddY mice maintained by Japan SLC (SLC, Shizuoka, Japan). All procedures relating to animal care and treatment conformed to the animal care guidelines of the Animal Experiment Committee of Gifu Pharmaceutical University. All efforts were made to minimize suffering and the number of animals used. The animals were housed at 24 ± 2°C under a 12 hr light-dark cycle (lights on from 8:00 to 20:00) and had ad libitum access to food and water.

### Chemicals and drugs

The following chemicals and drugs were used: Ethanol, hydrogen chloride, urethane, paraformaldehyde, indomethacin, ferrous sulfate (FeSO_4_), carboxymethylcellulose (CMC), and sodium chloride (WAKO Pure Chemical Industries, Osaka, Japan); perchloric acid (HClO_4_) and thiobarbituric acid (TBA) (Sigma, St. Louis, MO, USA); hydrochloride (HCl) (Nakarai Tesque, Ltd., Kyoto, Japan); and sodium dodecyl sulfate (SDS) (Life Technologies Japan Ltd., Tokyo, Japan). Sozinsan AT, gifted from Meiji Seika Kaisha, Ltd. (Tokyo, Japan), was used as the KRGCD. The KRGCD (1300 mg) contained 133.33 mg of KRG powdered extract, 200 mg of powdered Atractylodes lancea rhizome, 200 mg of powdered cinnamon bark, 400 mg of powdered oyster shell, 133.33 mg of magnesium hydroxide, 66.67 mg of Corydalis tuber powdered extract, and the appropriate amount of inactive ingredients. KRG was gifted from Nippon Funmatsu Yakuhin Co., ltd. (Osaka, Japan). Selbelle (Eisai Co., Ltd., Tokyo Japan) was used as the teprenone-containing drug (TCD). Storage (Takeda Pharmaceutical Co., Ltd., Osaka, Japan) was used as the Anchusan prescribed drug (APD).

### Hydrogen chloride (HCl)/ethanol-induced gastric ulcer

Gastric hemorrhagic lesions were induced by intragastric administration (0.1 ml/20 g) of an HCl/ethanol mixture, containing 150 mM HCl in 98% ethanol [17-19]. The KRGCD dissolved in CMC (0.5% carboxymethylcellulose solution dissolved in distilled water) was administered at oral doses of 30, 100, and 300 mg/kg, 1 hr before HCl/ethanol application. The TCD (76 mg/kg, p.o.)- and APD (96 mg/kg, p.o.)-treated groups were included as reference controls. We determined the doses of these control drugs in relation to 100 mg/kg of KRGCD, based on their defined daily dose for human (KRGCD: 3900 mg, TCD: 3000 mg, and APD: 3750 mg). One hr after ethanol administration, the animals were killed by cervical dislocation, and the stomachs were removed, inflated with 1 mL of 1% paraformaldehyde overnight to fix the tissue walls, and opened along the greater curvature. The ulcer lesions were stretched out, and software (Angiogenesis Image Analyzer, Kurabo, Osaka, Japan) was used to estimate their sizes.

### Indomethacin-induced gastric ulcer

Gastric hemorrhagic lesions were induced by intragastric administration (0.1 ml/10 g) of 20 mg/kg of indomethacin. KRGCD dissolved in CMC (0.5% carboxymethylcellulose solution dissolved in distilled water) was administered at oral doses of 30, 100, and 300 mg/kg, 1 hr before indomethacin application. The TCD (76 mg/kg, p.o.)- and APD (96 mg/kg, p.o.)-treated groups were included as the reference controls. We determined the doses of these control drugs in relation to 100 mg/kg of KRGCD, based on their defined daily dose for human (KRGCD: 3900 mg, TCD: 3000 mg, and APD: 3750 mg). Twelve hr after indomethacin administration, the animals were killed by cervical dislocation, and the stomachs were removed, inflated with 1 mL of 1% paraformaldehyde overnight to fix the tissue walls, and opened along the greater curvature. The hemorrhagic lesions were stretched out, and software (Angiogenesis Image Analyzer, Kurabo) was used to estimate their sizes.

### Histological analysis

Gastric tissue samples were fixed in neutral buffered formalin for 24 hr. Stomach sections were dehydrated with graded ethanol, passed through xylene, and embedded in paraffin. The paraffin sections (5 μm thick) were stained with hematoxylin/eosin (HE).

### Lipid peroxidation

In the in vitro model of lipid peroxidation, the supernatant fraction of a stomach homogenate from male adult ddY mice was prepared. Stomach tissues were homogenized in Hiscotron homogenizer in 5 vols of ice-cold 1.15% sodium chloride solution. Then, 945 μL of stomach homogenate was added to 5 μL of test compound and 50 μL of FeSO_4_, and incubated at 37°C for 1 hr. The reaction was stopped by adding 200 μL of 35% HClO_4_, and then centrifuged at 3,000 rpm for 10 min. The supernatant 500 μL was heated with 500 μL of TBA, 750 μL of phosphate buffer, and 100 μL of SDS for 60 min at 100°C. After being cooled, the reactants were supplemented with an equivalent amount of n-butanol, shaken vigorously for 1 min, and centrifuged for 10 min at 4,000 rpm. Absorbance was measured spectrophotometrically at 532 nm. In the ethanol-induced gastric ulcer model, mice were pretreated with CMC (p.o.) or KRGCD (30, 100, 300 mg/kg, p.o.) 1 hr before ethanol. At 1 hr after the application of ethanol, the animals were killed, and a glandular segment from each stomach was homogenized in 5 vols of ice-cold 1.15% sodium chloride solution, and the method described above was followed.

### Measurement of gastric mucosal blood flow

Gastric mucosal blood flow was measured using a laser Doppler flowmeter, FLO-N1 (Omegawave, Inc., Tokyo, Japan), as described in our previous reports [20]. The mice used for this measurement were anesthetized with urethane (1.2 g/kg, i.p.), and the abdomen was opened on an operation mat. The mat was heated at 37°C during the operation and blood flow measurement. Then, a non-contact probe was placed gently 1.0 mm above and perpendicular to the mucosal surface in the pylorus area to monitor gastric mucosal blood flow. After the gastric mucosal blood flow was stable, saline, KRGCD (100 and 300 mg/kg, i.g.), and KRG (30 and 100 mg/kg, i.g.), which dissolved in saline, were administrated directly into the stomach with a cannula, and gastric mucosal blood flow was monitored for 1 hr.

### Statistical analysis

Data are presented as the means ± standard error of the mean (SEM). Statistical comparisons were made with Student's *t*-test or one-way analysis of variance (ANOVA) followed by Dunnett's test using Statview version 5.0 (SAS Institute Inc., Cary, NC, USA), with p < 0.05 being considered to indicate a statistical significance.

## Results

### Effect of KRGCD on HCl/ethanol-induced gastric damage

**Figure 1:**
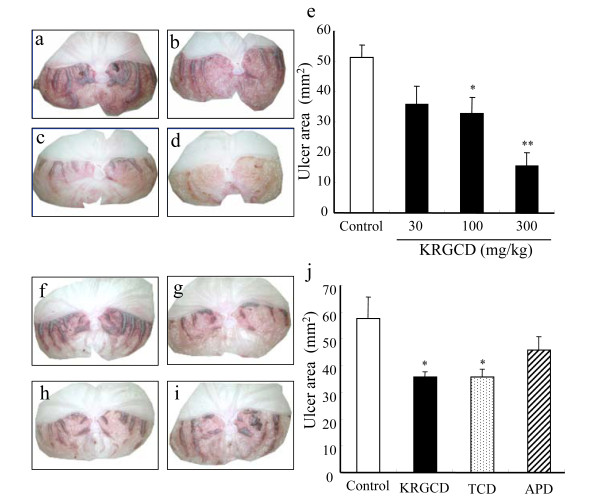
**Protective effect of KRGCD on gastric mucosal lesions induced by HCl/ethanol in mice**. Stomachs from mice with vehicle (a) and pretreated with KRGCD at doses of 30 mg/kg (b), 100 mg/kg (c), and 300 mg/kg (d) at 1 hr before HCl/ethanol administration. Quantitative analysis of KRGCD on the ulcer area (mm^2^) induced by HCl/ethanol (e). n = 13 for each group. Each value represents the mean ± SEM. * p < 0.05, ** p < 0.01 vs. control mice, Dunnett's multiple-range test. Stomachs from mice with vehicle (f) and pretreated with KRGCD (100 mg/kg) (g), TCD (76 mg/kg) (h), and APD (96 mg/kg) (i) at 1 hr before HCl/ethanol administration. Quantitative analysis of KRGCD, TCD, or APD on ulcer area (mm^2^) induced by HCl/ethanol (j). n = 8 for each group. Each value represents the mean ± SEM. * p < 0.05 vs. control mice, Student's *t*-test.

The ulcer-preventive effect of KRGCD was evaluated by using ethanol (containing 150 mM of hydrochloride)-induced ulcers. Ethanol induced intense gastric mucosal damage in the form of hemorrhagic streaks in the control group of mice that received vehicle alone (Figure 1a and 1e). Oral treatment of 100 and 300 mg/kg of KRGCD significantly reduced the gastric mucosal damage and quantitative reduction in a dose-dependent manner compared with the control-treated group (Figure 1b, c, d, and 1e). Compared with the reference drugs, which were used at a dose equivalent to 100 mg/kg of KRGCD, KRGCD is equally efficacious in the treatment of TCD (76 mg/kg, p.o.) (Figure 1g, h, and 1j). APD (96 mg/kg, p.o.) also showed a tendency to protect against ethanol-induced gastric damage, but the tendency was not significant (Figure 1i and 1j).

### Effect of KRGCD on indomethacin-induced gastric damage

**Figure 2:**
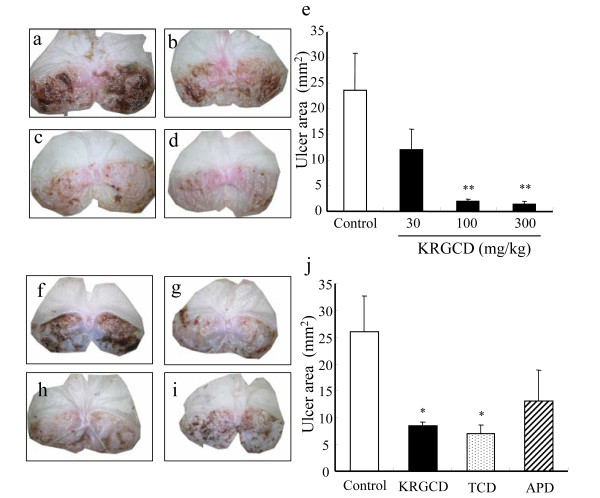
**Protective effect of KRGCD on gastric mucosal lesions induced by indomethacin in mice**. Stomachs from mice with vehicle (a) and pretreated with KRGCD at doses of 30 mg/kg (b), 100 mg/kg (c), and 300 mg/kg (d) at 1 hr before indomethacin administration. Quantitative analysis of KRGCD on the ulcer area (mm^2^) induced by indomethacin (e). n = 6 for each group. Each value represents the mean ± SEM. ** p < 0.01 vs. control mice, Dunnett's multiple-range test. Stomachs from mice with vehicle (f) and pretreated with KRGCD (100 mg/kg) (g), TCD (76 mg/kg) (h), and APD (96 mg/kg) (i) at 1 hr before indomethacin administration. Quantitative analysis of KRGCD, TCD, or APD on the ulcer area (mm^2^) induced by indomethacin (j). n = 8 for each group. Each value represents the mean ± SEM. * p < 0.05 vs. control mice, Student's *t*-test.

The gastroprotective effect of KRGCD on indomethacin-induced gastric damage was macroscopically determined in mice. Macroscopic lesions with evident borderlines in various forms and sizes were dispersed irregularly on all stomach surfaces in the stomach tissue of the control mice that received indomethacin (Figure 2a). The mice that received oral treatment of 100 and 300 mg/kg of KRGCD showed gastric mucosal protection and quantitative reduction in a dose-dependent manner compared with the control-treated group (Figure 2b, c, d, and 2e). Compared with the reference drugs, which were used at a dose equivalent to 100 mg/kg of KRGCD, KRGCD is equally efficacious in the treatment of TCD (76 mg/kg, p.o.) (Figure 2g, h, and 2j). APD (96 mg/kg, p.o.) also showed a tendency to protect against indomethacin-induced gastric damage, but the tendency was not significant (Figure 2i and 2j).

### Effect of KRGCD on histological evaluation in an HCl/ethanol-induced ulcer model

**Figure 3:**
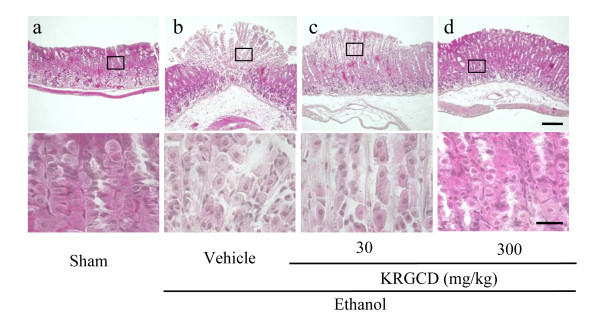
**Effect of KRGCD on histological evaluation in an HCl/ethanol-induced ulcer model**. Sections of hematoxylin and eosin (HE) staining (a-d). Section through gastric mucosa in the sham treated group (a). Microscopic appearance of lesions induced by HCl/ethanol in gastric mucosa pretreated with vehicle (b). Microscopic appearance of lesions pretreated with 30 mg/kg of KRGCD (c). Microscopic appearance of lesions pretreated with 300 mg/kg of KRGCD (d). Scale bar = 150 μm (upper micrograph) and 30 μm (lower micrograph).

Histopathological studies further confirmed that pretreatment with KRGCD prevented ethanol-induced histological damage in the superficial layers of the gastric mucosa with congestion by HE staining. Compared with the sham operating group, ethanol administration induced a disruption of the superficial region of the gastric gland with epithelial cell loss and intense edema formation (Figure 3a and 3b). Histological analysis showed that KRGCD (300 mg/kg, p.o.) prevented this damage (Figure 3d), but not 30 mg/kg administration (Figure 3c). KRGCD (100 mg/kg, p.o.)-treated group showed similar staining pattern to 300 mg/kg-treated group (data not shown).

### Effect of KRGCD on histological evaluation in an indomethacin-induced ulcer model

**Figure 4:**
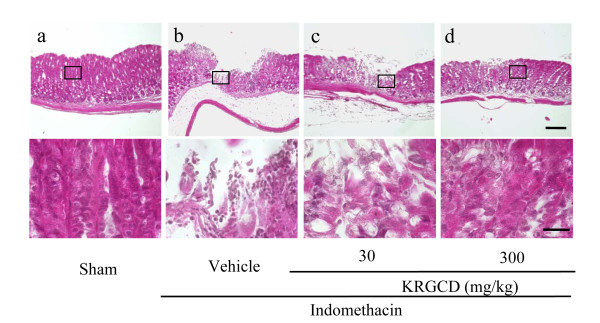
**Effect of KRGCD on histological evaluation in indomethacin-induced ulcer model**. Sections of hematoxylin and eosin (HE) staining (a-d). Section through gastric mucosa in the sham treated group (a). Microscopic appearance of lesions induced by indomethacin in gastric mucosa pretreated with vehicle (b). Microscopic appearance of lesions pretreated with 30 mg/kg of KRGCD (c). Microscopic appearance of lesions pretreated with 300 mg/kg of KRGCD (d). Scale bar = 150 μm (upper micrograph) and 30 μm (lower micrograph).

Histopathological studies further confirmed that pretreatment with KRGCD prevented indomethacin-induced histological damage in the superficial layers of the gastric mucosa with congestion by HE staining. Compared with the sham operating group, indomethacin administration induced a disruption of the superficial region of the gastric gland with epithelial cell loss (Figure 4a and 4b). Histological analysis showed that 300 mg/kg of KRGCD (p.o.) prevented this damage (Figure 4d), but not 30 mg/kg (Figure 4c). KRGCD (100 mg/kg, p.o.)-treated group showed similar staining pattern to 300 mg/kg-treated group (data not shown).

### Effect of KRGCD on lipid peroxidation

**Figure 5:**
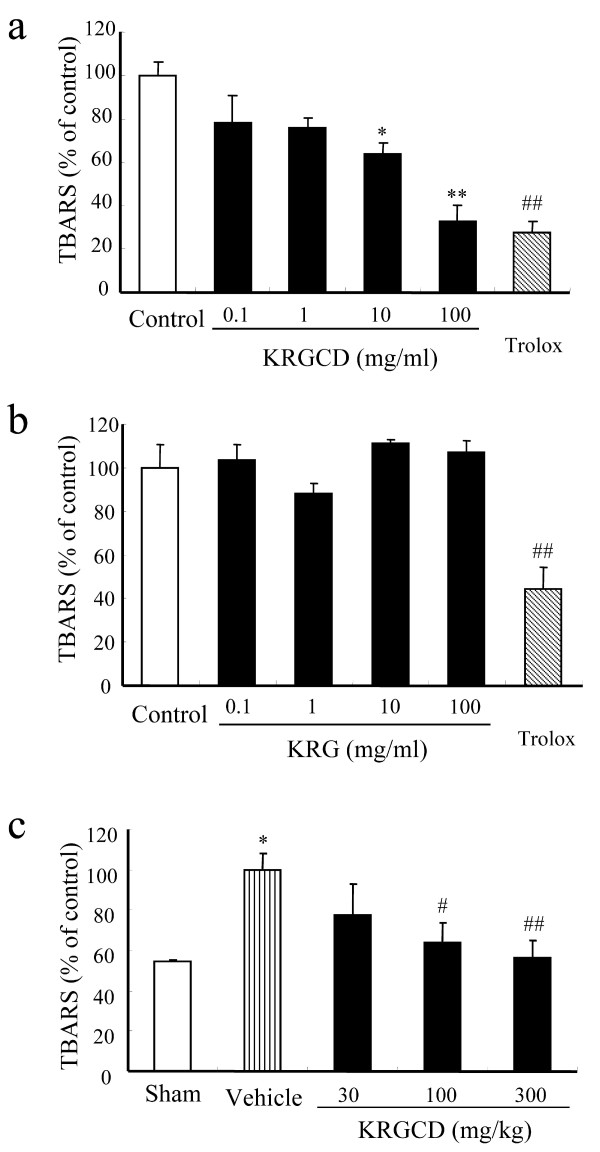
**Effect of KRGCD on lipid peroxidation**. Effects of KRGCD (a) and KRG (b) on TBARS content in in vitro mouse stomach homogenates. n = 4 for each group. Each value represents the mean ± SEM. * p < 0.05, ** p < 0.01 vs. control, Dunnett's multiple-range test. ^## ^p < 0.01 vs. control, Student's *t*-test. Effect of KRGCD (c) on TBARS content in mice on HCl/ethanol-induced gastric damage. Sham (n = 3), control (n = 10), 30 mg/kg of KRGCD (n = 6), 100 mg/kg of KRGCD (n = 8), and 300 mg/kg of KRGCD (n = 8). * p < 0.05, vs. sham treated group, Student's *t*-test. ^# ^p < 0.05, ^## ^p < 0.01 vs. control, Dunnett's multiple-range test.

Gastric ulcer is well-accepted oxidative stress-induced stomach disease, and the mechanism of ulcer induction is mediated by reactive oxygen species (ROS). In vitro lipid peroxidation levels in the mice stomach homogenate was measured as TBARS used as an index of lipid peroxidation. The TBARS level increased after 1-hr incubation with FeSO_4 _at 37°C. KRGCD significantly inhibited the production of the TBARS level in a concentration-dependent manner (Figure 5a). The antioxidant trolox (100 μM; a water-soluble vitamin E derivative) also inhibited the production of TBARS. On the other hand, KRG did not decrease the production of TBARS in the mice stomach homogenate (Figure 5b). Next, we investigated the anti-oxidative effects of KRGCD in the in vivo model of an ethanol-induced gastric ulcer. Ethanol significantly enhanced the TBARS level compared to the values seen in the sham treating group; pretreatment of animals with KRGCD (100 and 300 mg/kg, p.o.) resulted in marked suppression of ethanol-induced TBARS (Figure 5c), indicating that this agent suppressed lipid peroxidation.

### Effect of KRGCD and KRG on gastric mucosal blood flow

**Figure 6:**
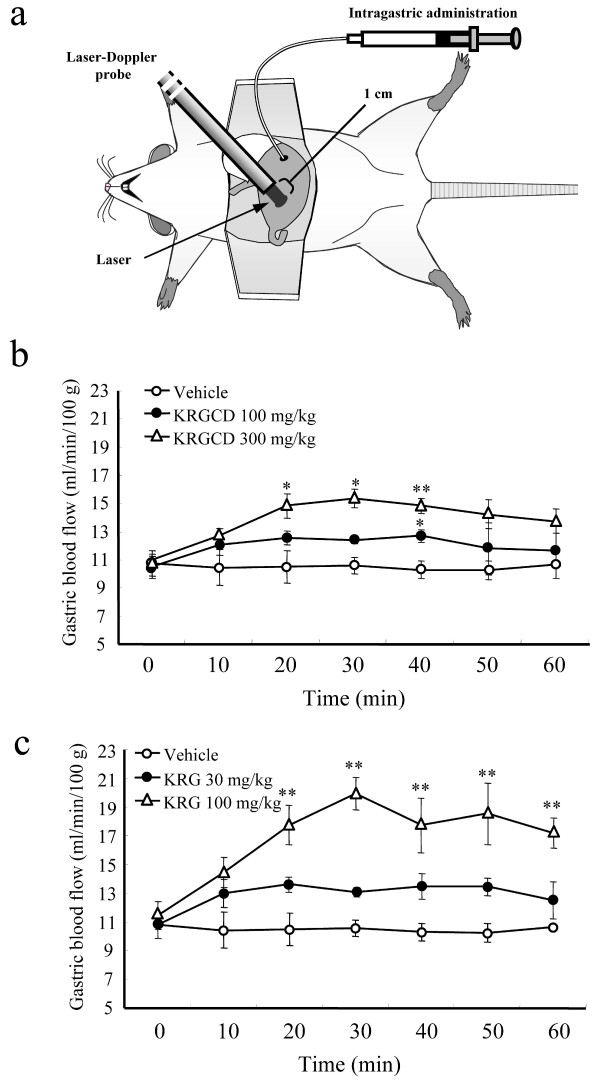
**Effects of KRGCD and KRG on the gastric mucosal blood flow**. Gastric mucosal blood flow was measured using a laser Doppler flowmeter (a). Effects of KRGCD (b) and KRG (c) on basal gastric mucosal blood flow. n = 3-4 for each group. Each value represents the mean ± SEM. * p < 0.05, ** p < 0.01 vs. control, Dunnett's multiple-range test.

Gastric mucosal blood flow plays an important role in gastric ulcer pathogenesis and healing. We investigated the effects of KRGCD and KRG on basal gastric blood flow in mice. KRGCD (100 and 300 mg/kg, i.g.) significantly increased the gastric mucosal blood flow in a time-dependent manner; the effects reached a peak at 30-40 min after KRGCD administration (Figure 6b). KRG (100 mg/kg, i.g.) also increased the gastric mucosal blood flow in a time-dependent manner (Figure 6c).

## Discussion

In this study, the anti-ulcer effect of KRGCD was investigated in mice using HCl/ethanol and indomethacin-induced ulcer models. These two models are the most commonly employed tests in evaluations of anti-ulcer activity. In addition, the effects of KRGCD on anti-oxidant activity and gastric mucosal blood flow in mice stomach tissue were evaluated.

KRGCD was found to significantly inhibit HCl/ethanol- and indomethacin-induced ulcers, and the anti-ulcer capacity of KRGCD was determined to be dose-dependent. Ethanol induces severe gastric mucosal damage in humans and rodents [10-12] and rapidly penetrates the gastric mucosa, causing membrane damage, exfoliation of cells, and erosion. The subsequent increase in mucosal permeability together with the release of vasoactive products from mast cells, macrophages, and other blood cells can lead to vascular injury, necrosis, and ulcer formation [13].

Oxidative stress is believed to initiate and aggravate many digestive system diseases, including stomach ulcers and gastric carcinoma. Especially, ethanol-induced gastric damage has been suggested to be mediated by the generation of free radicals [21]. Ethanol is metabolized in the body and releases superoxide anion and hydroperoxy free radicals. As previously reported, oral administration of HCl/ethanol significantly increased the lipid peroxidation compared with the sham treating group, and KRGCD significantly reduced the production of TBARS in a concentration-dependent manner. However, KRG did not attenuate the production of TBARS in the mice stomach homogenate. These results suggest that the anti-oxidative activity of KRGCD is not due to KRG but to other components in KRGCD, such as Cinnamomi cortex and Atractylodes lancea rhizome. Cinnamomi cortex has been reported to contain very high concentrations of antioxidants [22]. Toki-shakuyaku-san, a kind of traditional Chinese medicine containing Atractylodes lancea rhizome, has also been reported to possess a potent anti-oxidative effect [23].

In this study, to consider the influence of another vegetal drugs containing in KRGCD, we evaluated Teprenon-containing drug (TCD) and Anchusan-prescribed drug (APD) as positive controls. Among the vegetal drugs in KRGCD, TCD contains Atactylodes lancea rhizome and Cinnamon bark. APD also contains Cinnamon bark, Oyster shell, and Corydalis tuber. Because TCD had a similar beneficial effect to KRGCD against ethanol and indomethacin induced gastric ulcer, Atactylodes lancea rhizome which containing both in KRGCD and TCD, not in APD, may contribute to the potent gastroprotective effect of KRGCD. On the other hand, as the other vegetal drugs, such as Cinnamon bark [24] and Oyster shell [25], are reported to have a protective effect against gastric ulcer or gastric mucosal injury, these components may contribute to the effect of KRGCD. However, considering the high KRG contents in KRGCD as original natural medicine, KRG may play a prominent role in gastroprotective effects of KRGCD.

Another important finding in our study is that KRGCD and KRG administration enhances basal gastric mucosal blood flow in intact mice. A previous study has also shown that KRG improves the systemic blood flow, such as liver, spleen, kidney, and gastric mucosa in rats, using the hydrogen gas clearance method [26]. In the current study, we confirmed the effect in mice. A laser Doppler flowmeter with a non-contact probe allowed us to measure the gastric mucosal blood flow stably in mice. In figure 3b, KRGCD at 300 mg/kg increased the gastric mucosal blood flow to around 15 (ml/min/100 g). In figure 3c, KRG at 30 mg/kg, which is in relation to 300 mg/kg of KRGCD, also increased the flow to around 13.5 (ml/min/100 g). Although almost similar rate of increase was obtained between two groups, the effect of KRGCD on gastric mucosal blood flow was slightly higher than that of KRG. These results indicate that the other vegetal drugs containing in KRGCD, such as Cinnamon bark [24], may in part contribute to the effect of KRGCD on the gastric mucosal blood flow. Sufficient gastric mucosal blood flow is important for maintaining normal secretion, digestion, and motility of stomach and plays an important role in the development of ulcers [27]. To protect the gastric mucosa, a complex defense system, which includes the production of surface mucus and bicarbonate and the regulation of gastric mucosal blood flow, has evolved. The main factors, which regulate gastric blood flow, are prostaglandins, sensory peptides released from endings of afferent nerves, and nitric oxide (NO) [28,29]. In a previous study, saponin fraction of KRG increased the cerebral blood flow [30] and increased the level of nitric oxide in the endothelial cells [31]. It has been also shown that ginseng saponin relaxed the basilar artery in a dose-dependent and partly endothelium-dependent manner [32]. Therefore, our results suggest that KRG increased the gastric mucosal blood flow, perhaps due to the vasorelaxing action of KRG in the gastric mucosal vessel in the resting state. On the other hand, the drug has a possibility to induce astriction and diarrhea as a side effect (information from company).

## Conclusions

KRGCD showed protective effects on HCl/ethanol- and indomethacin-induced gastric mucosal injury in a dose-dependent manner. These results lead us to believe that KRG and its prescription may be a powerful remedy for gastric mucosal lesions, inhibiting lipid peroxidation and enhancing gastric mucosal blood flow.

## Abbreviations

APD: Anchusan-prescribed drug; CMC: Carboxymethylcellulose; HE: Hematoxylin/eosin; KRG: Korean red ginseng; KRGCD: Korean red ginseng-containing drug; TCD: Teprenone-containing drug

## Competing interests

The authors declare that they have no competing interests.

## Authors' contributions

AO, KO, and MK performed the study. AO wrote the paper. HH participated in the design of the study. All authors read and approved the final manuscript.

## Pre-publication history

The pre-publication history for this paper can be accessed here:

http://www.biomedcentral.com/1472-6882/10/45/prepub
